# The requirements for rumen-degradable protein per unit of fermentable organic matter differ between fibrous feed sources

**DOI:** 10.3389/fmicb.2015.00715

**Published:** 2015-07-14

**Authors:** Carla R. Soliva, Sergej L. Amelchanka, Michael Kreuzer

**Affiliations:** ^1^Agrovet-Strickhof, ETH ZurichZurich, Switzerland; ^2^Institute of Agricultural Sciences, ETH ZurichZurich, Switzerland

**Keywords:** ammonia, *brachiaria*, sugar beet pulp, apple pomace, nitrogen

## Abstract

Ruminant feed evaluation systems use constant minimum requirements of rumen-degradable protein (RDP) and often relate this to apparently degradable organic matter (OM). However, studies with tropical forages indicate that RDP: apparently degraded OM might not be constant across high-fiber diets. This was tested with semi-continuous ruminal cultures (Rusitec) using dried contrasting low-protein fiber sources: brachiaria hay (high in fiber, medium lignified), apple pomace (medium in fiber, highly lignified), and sugar beet pulp (medium in fiber and lignification). Each feed was incubated at 14 g dry matter day^−1^ with 0, 0.85, 1.7, 3.4, 6.8, 13.6, or 27.2 mg g^−1^ urea. The amount of urea needed to reach a similar basal concentration of ammonia in the incubation fluid was tested for each feed in advance. Apparent fiber and OM degradability were determined after 48 h of incubation. Data was evaluated by regressions and analysis of variance. The response curve of incubation fluid ammonia to urea supplementation was similar in slope in all feeds. Plateaus in apparent OM degradability in relation to ammonia concentration were determined. The ammonia concentration where apparent OM and fiber degradability reached 95% of maximum was approached in the order of pomace < pulp < hay. With regard to fiber degradability, a plateau was reached at ≥ 80 g kg^−1^ crude protein only with hay and pomace, whilst a linear relationship existed between RDP and OM degradation for pulp. In hay the ratio RDP: OM degraded was equal to 1.6 but was only 1.0 in the other feeds. There was no obvious lack of branched short-chain fatty acids at low RDP. Thus, the hypothesis was confirmed but the demand for RDP seems even higher in tropical forage compared to food industrial byproducts. The efficiency of urea to promote apparent OM and fiber degradation was also variable. Thus, it seems that minimum thresholds of either RDP or ruminal ammonia concentration may not be reflected appropriately by constants.

## Introduction

Various tropical grass species and harvest residues are very low in crude protein (CP) and RDP (e.g., Mathis et al., 2000; Schwab et al., 2005). Especially during the dry season, tropical forages have CP values lower than 70 g kg^−1^ dry matter (DM), which is considered limiting for the maintenance of an adequate ruminal fibrolytic activity (Lazzarini et al., 2009; Sampaio et al., 2009). Such sub-optimal ruminal conditions would be associated with low microbial growth and reduced fiber degradation (Sampaio et al., 2010). Increasing the dietary CP concentration close to 100 g kg^−1^ of DM was found by Sampaio et al. (2010) to optimize the use of low-quality tropical forage; ruminal ammonia N concentrations of 3.6–7.2 mmol l^−1^ were therefore considered supplying enough nitrogen to maintain the microbial activity in the rumen. In general, the high importance of RDP as nutrient for fiber degrading microbes in the rumen is known, and most feed evaluation systems for ruminants specify a minimum threshold for dietary RDP (60–80 g kg^−1^ DM) or CP (ranging from >120 to 130 to >160 to 180 g kg^−1^ DM) (reviewed by Schwab et al., 2005). Another approach for determining requirements for RDP is oriented toward minimum rumen fluid ammonia concentrations. Here the recommended concentrations vary extremely, namely between >1.2 (Marini and Van Amburgh, 2003) and >16 mmol l^−1^ (Mehrez et al., 1977). Even when aligning this with the low CP concentrations typical for tropical forages, such forages still were often found to be sufficient to cover at least maintenance requirements of livestock for nitrogen. Consistent with this, feeding tallgrass-prairie hay to cows, containing only 19 g CP kg^−1^ DM, still yielded a digestibility of the neutral detergent fiber (NDF) of 47%, and digestibility was only increased to on average 55% with additional supplemented RDP ranging from 180 to 720 g DM d^−1^ compared to the not RDP-supplemented treatment (Köster et al., 1996). Also, an acceptable NDF digestibility of 59% was found for sheep consuming only brachiaria hay, a wide-spread cultivated tropical grass, with 61 g CP kg^−1^ DM; this, however, at a ruminal ammonia concentration of 3.9 mmol l^−1^ (Tiemann et al., 2008b). Also the fiber of hay prepared from *Brachiaria* containing <40 g CP kg^−1^ DM, was found to be reasonably well fermented in the Rumen Simulation Technique (Rusitec) despite extremely low ammonia concentrations of 0.3–0.6 mmol l^−1^ found in the incubation fluid (Hess et al., 2003; Tiemann et al., 2008a).

Overall, these findings might suggest that less RDP is required to ferment OM and fiber of low-CP (tropical) forages although, based on theoretical considerations, the ratio of RDP: apparently degraded OM needed by the ruminal microbes should be rather static across fibrous feeds sources (Bach et al., 2005). This would imply that RDP requirements might also differ among feeds with similar digestibility, an assumption that goes beyond the known phenomenon that the required concentrations of either ruminal ammonia or RDP differ for feeds with varying digestibility only (e.g., Erdmann et al., 1986; Odle and Schaefer, 1987). Also, the NRC (2001) states, that the requirement for RDP depends on the fermentability of the basal diet. When slowly fermentable roughages are fed, reduced amounts of RDP would be required, whereas when excessive amounts of degradable protein is fed, N losses from the rumen and decreased capacity to recycle N will likely occur (Siddons et al., 1985). A clear indication that the RDP: apparently degraded OM ratio, i.e., the requirement for RDP, could vary among low-CP forages was described by Mathis et al. (2000) comparing the results of three experiments with steers each carried out with different low-CP forages. These forages included Bermuda grass (CP 82 g kg^−1^ DM; RDP 48 g kg^−1^ DM), bromegrass (CP 59 g kg^−1^ DM, RDP 29 g kg^−1^ DM), and forage sorghum (CP 43 g kg^−1^ DM, RDP 25 g kg^−1^ DM). In that study, the proportion of RDP in digestible OM varied for each of the forages between 8.2 and 16.2% for Bermuda grass and from 5.5 and 6.0 to 12.4 and 12.8% for bromegrass and forage sorghum, respectively. Mathis et al. (2000) did not specify the degree of lignification of the forages, and differences were explained by the authors as being an effect of animal-individual concentrations of N recycling within the animals. Still, other factors might be equally important like the cellulose-to-hemicellulose ratio (Griswold et al., 2003) or other fiber properties.

The hypotheses to be tested in the present *in vitro* study were that (i) the requirements for RDP per unit of degradable OM are not static across forages differing in fiber concentration and degree of lignification, and that (ii) differences in RDP requirements per unit of OM degraded are not only the result of variations in N recycling via saliva. For that purpose, the continuously operating Rusitec was employed, a technique excluding the influence of postruminal digestion of OM and fiber as well as of N recycling. Fiber sources were selected which guaranteed a large variation in fiber composition and that were low in crude protein (RDP) to be able to generate low-RDP treatments. In order to obtain information about the status of supply of the ruminal microbes with branched-chain carbon sources under different RDP supply, special attention was paid to the concentration of branched short-chain fatty acids (SCFA) in incubation fluid.

## Materials and methods

### Experimental feeds

Three low-protein fibrous feeds, largely differing in composition of cell wall constituents, were selected for the present study: hay from a tropical grass species (*Brachiaria brizantha* cv. Toledo; CIAT accession no. 26110, Cali, Colombia; high in fiber, medium lignified), obtained from the same batch of hay used in a previous experiment with sheep (Tiemann et al., 2008b), and two dried food industrial by-products, apple pomace (medium in fiber, highly lignified), and sugar beet pulp (medium in fiber and lignification). The analyzed nutrient composition (Table 1) slightly differed from that reported for the pooled brachiaria samples across the entire sheep experiment (Tiemann et al., 2008b).

**Table 1:** **Analyzed nutrient composition and fermentation characteristics (average of incubation days 6 through 10) of the three experimental feeds (*n* = 7; i.e., across all urea supplementations)^1^**.

	**Brachiaria hay**	**Apple pomace**	**Beet pulp**	**SEM^2^**	***P*-value**
**NUTRIENTS [g kg^−1^ DRY MATTER (DM)]**					
DM (g kg^−1^ feed as used)	924	913	911		
Organic matter (OM)	920	989	945		
Crude protein	69	52	91		
Neutral detergent fiber (NDF)	669	474	430		
Acid detergent fiber (ADF)	402	333	234		
Acid detergent lignin (ADL)	55	167	77		
Hemicellulose (NDF-ADF)	267	141	196		
Cellulose (ADF-ADL)	347	166	157		
**INCUBATION FLUID PH AND MICROBIAL COUNTS**					
Incubation fluid pH	6.85^a^	6.86^a^	6.54^b^	0.043	<0.001
Total bacteria (10^8^ ml^−1^)	2.00^a^	1.13^b^	1.98^a^	0.095	<0.001
Total protozoa (10^3^ ml^−1^)	1.05^b^	3.05^a^	0.91^b^	0.413	<0.001
**SHORT-CHAIN FATTY ACIDS**					
Total SCFA (mmol day^−1^)	38.5^ab^	30.7^b^	48.8^a^	4.03	0.001
Acetate (%)	65.2^a^	59.2^b^	63.2^a^	1.28	<0.001
Propionate (%)	23.5	22.4	22.8	0.81	0.611
*n*-butyrate (%)	10.0^b^	16.2^a^	11.8^b^	1.27	<0.001
*iso*-butyrate (%)	0.283^ab^	0.520^a^	0.203^b^	0.0922	0.022
*n*-valerate (%)	0.71^b^	1.23^b^	1.87^a^	0.248	<0.001
*iso*-valerate (%)	0.263^ab^	0.484^a^	0.179^b^	0.0843	0.014
Acetate: propionate ratio	2.79	2.68	2.79	0.337	0.782
**APPARENT DEGRADABILITY (%)**					
OM	40.9^b^	35.6^b^	55.8^a^	2.12	<0.001
NDF	36.0^b^	19.3^c^	47.6^a^	2.31	<0.001
ADF	32.4^a^	5.3^b^	38.3^a^	2.41	<0.001
**RUMEN-DEGRADABLE PROTEIN (RDP) REQUIRED (g g^−1^ FERMENTED NUTRIENTS)**					
OM	1.63^a^	1.08^b^	1.00^b^	0.308	<0.001
NDF	1.87^a^	2.15^a^	1.18^b^	0.493	<0.001
ADF	2.09	5.01	1.49	3.954	0.173
**METHANE**					
ml day^−1^	127	110	197	21.0	0.055
ml g^−1^ degraded OM	24.6	23.4	26.5	3.00	0.906
ml g^−1^ degraded NDF	33.3^b^	71.3^a^	58.6^ab^	7.17	0.013

1 *Not including treatment with lowest urea supplementation, i.e., 0.42 g kg^−1^ dietary DM, with apple pomace. Means within the same row carrying no common superscript differ at P < 0.05*.

2 *SEM, standard error of the mean*.

### *In vitro* technique

For the present study, an eight-fermenter Rusitec was used. The construction and operation of this equipment is described in detail in Soliva and Hess (2007). The daily portions (14 g of DM) of the experimental feeds, ground to pass a 5-mm sieve, were put into nylon bags (70 × 140 mm) with a pore size of 100 μm. Urea was continuously infused into the fermenters after being mixed with the respective buffer solution following the approach of Griswold et al. (2003). Individual buffer storage canisters were used for each fermenter, but buffer flow rate to the fermenters was kept constant at 435 ± 23 ml day^−1^ for all fermenters. The rumen fluid originated from a non-lactating rumen-cannulated Brown Swiss cow that received temperate climate grass hay at *ad libitum* access and about 1 kg d^−1^ of concentrate. The cow was treated in accordance to the Swiss guidelines for animal welfare. For each experimental run, incubations lasted for 10 days with day 6 through day 10 being used for data evaluation. Following this adaptation scheme also ensured that incubation fluid was depleted of the nitrogen compounds initially introduced into the system through the rumen fluid.

### Preliminary assessments

The N source of choice for the present study was urea, as it is completely degradable. However, low-quality highly fibrous roughages may be limited with respect to ruminal fermentation in more than only a lack of degradable N. Therefore, two 10-day preliminary experimental assessments using Rusitec were carried out prior to the main experiment. The first preliminary assessment was carried out to evaluate whether or not fiber degradation by the ruminal microbes in Rusitec is improved by the supplementation of urea and specific microbial growth factors. In addition, changes in incubation fluid ammonia concentration due to urea supplementation were recorded. In the second preliminary assessment the change in incubation fluid ammonia concentration in response to urea supplementation was recorded when incubating the three experimental feeds selected (brachiaria hay, apple pomace, and beet pulp). This was done to be able to calculate the urea amounts required for balancing differences in ammonia concentration between feeds.

In the first assessment, five treatments tested in one experimental run (*n* = 1), all based on 15 g DM day^−1^ of brachiaria hay but differing in the supplementation of urea and microbial growth factor solution (GF), were tested with Rusitec by providing per g hay DM: (1) zero supplementation; (2) 0.13 ml GF; (3, 4) 4 mg urea, without or with 0.13 ml GF; and (5) 30 mg urea + 0.13 ml GF. The growth factor solution was composed of (μg ml^−1^) acetate, 1330; iso-butyrate, 66; iso-valerate, 80; valerate, 80; pyridoxine, 2; thiamine, 2; biotin, 0.05; 4-aminobenzoic acid, 0.1 (as described by Scott and Dehority, 1965). The resulting change in the extent of NDF degradability within 48 h of incubation occurring with increasing urea supplementation was as expected (23.9, 27.7, and 36.2% with urea supplementations at 0, 4, and 30 mg g^−1^ feed DM). The addition of the growth factor solution when provided without (23.9 vs. 20.9%, treatment 1 vs. 2) or together with the low urea supplementation (27.6 vs. 27.6%, treatment 3 vs. 4) did not noticeably modify NDF degradability. These findings suggested that, under the present experimental conditions, the rumen fluid used was sufficiently rich in potentially limiting microbial growth factors other than RDP, and no addition of the growth factor solution was required to allow that effects of RDP were fully expressed. Potential limitations given by omitting the addition of preformed, mostly branched-chain AA (Owens and Bergen, 1983) were not tested.

The second assessment, based on the results of the first preliminary assessment and previous, unpublished data, aimed at achieving the same target incubation fluid ammonia concentration of 1.22 mmol l^−1^ for all three feeds (i.e., the basal concentration found with beet pulp), 19.52 mmol l^−1^ for beet pulp and apple pomace (as the concentration approximating the highest one aimed at in the main experiment), and additionally 1.84 mmol l^−1^ for beet pulp (reflecting an intermediate target). The latter two aimed to match the differing recommended minimum rumen fluid ammonia concentrations of >1.2 and >16 mmol l^−1^ specified by Marini and Van Amburgh (2003) and Mehrez et al. (1977), respectively. Without supplementation, the ammonia concentrations assumed from previous, unpublished incubation data were 0.83 and 0.03 mmol l^−1^ for brachiaria hay and apple pomace, respectively. Each of the treatments was tested in the same experimental run (*n* = 1). For the first 5 days of incubation, amounts of urea supplementation were fixed in advance with the goal to add urea in amounts necessary to balance for the differences in ammonia concentrations of the non-supplemented fiber sources. This first attempt did not result in a fully balanced ammonia concentration. Therefore, fine-tuning was performed for the second half of the 10-day incubation period. Apart from the zero control without urea, this finally meant urea supplementations (g kg^−1^ feed DM) of 0.6 for brachiaria hay, 1.3 and 39.0 for beet pulp, and 2.6 and 41.5 for apple pomace. Although the maximum target ammonia concentrations had been approximately reached on average (e.g., maximum values of 18 and 21 mmol ammonia l^−1^ with the high amount of urea in apple pomace and beet pulp, respectively), it was not possible to generate ammonia concentrations being stable across days of incubation. Therefore, for the main experiment it was decided to use the same fixed urea concentrations for strategic increase of incubation fluid ammonia concentrations for all three feeds. Additionally it was considered sufficient to balance only between the feed with the lowest CP concentration, apple pomace, and the other two feeds for the low level control treatments since the latter two feeds had yielded quite similar ammonia concentrations without extra urea (0.9 and 0.7 mmol l^−1^ in brachiaria hay and beet pulp, respectively) even though beet pulp had a higher CP concentration as brachiaria hay.

### Main experiment

The fibrous feeds were tested together with various amounts of urea supplementation. These treatments were randomly allocated to the eight fermenters in three experimental runs (*n* = 1). Urea supplementations chosen were always doubling the previous value with 0.85, 1.7, 3.4, 6.8, 13.6, and 27.2 g kg^−1^ dietary DM. The latter was approximately equivalent to the maximum amount still considered safe, because an urea supplementation level as high as 25 g kg^−1^ DM has been shown previously to be without adverse effects on cattle performance (Duff et al., 2003). Additionally, all feeds were incubated without urea supplementation and an additional urea supplementation was tested with apple pomace at a level of 0.42 g kg^−1^ DM following the decisions made from the results of the second preliminary assessment. Each treatment was tested once in order to generate a regression type of experimental design. The zero urea supplementation treatment with brachiaria hay, however, was repeated in each of the three experimental runs and reflected the control. These data were used to adjust all data across runs based on the calculated proportionate deviations of the control data obtained in the first two runs from the third experimental run. These adjustments mostly did not diverge from the unadjusted values by more than 2–4%.

### Laboratory analysis

Incubation fluid samples were analyzed daily for redox potential (for confirmation of the presence of anaerobic conditions), pH and ammonia concentration using the respective electrodes connected to a pH meter (model 634; Metrohm AG, Herisau, Switzerland). Counts of ciliate protozoa and total bacteria in the incubation fluid were determined as described by Soliva and Hess (2007) daily from experimental days 6–10 by using 0.1- and 0.02-mm depth Bürker counting chambers (Blau Brand, Wertheim, Germany), respectively. Prior to counting, microbes were fixed by the addition of 0.1 ml/ml (protozoa) and 0.99 ml ml^−1^ (bacteria) of Hayem solution (mg ml^−1^, HgCl_2_, 2.5; Na_2_SO_4_, 25.0; NaCl, 5.0). Incubation fluid samples were centrifuged for 5 min at 4000 g (Varifuge® K, Heraeus, Osterode, Germany) and the supernatant was stored at −20°C before being analyzed for the concentration of SCFA using HPLC (System Hitachi Lachrom, Merck, Tokyo, Japan) following the procedure of Ehrlich et al. (1981). After removal from the fermenters, the nylon bags with residues were washed in cold water in a washing machine to remove nitrogen containing particles originating from ruminal microbes and frozen at −20°C. Later they were lyophilized, weighed, and ground to pass a 1-mm sieve. Feeds and fermentation residues were analyzed following standard protocols (AOAC, 1997). They were analyzed for DM and total ash (TGA-500, Leco Corporation, St. Joseph, Michigan, USA), and N (C/N analyzer, Leco-Analysator Typ FP-2000, Leco Instrumente GmBH, Kirchheim, Germany; with CP defined as 6·25 × N). Further, NDF, acid detergent fiber (ADF), and acid detergent lignin (ADL) (Tecator Fibertec System M, 1020 Hot Extraction, Höganäs, Sweden) were analyzed, with NDF being determined with α-amylase addition but without sodium sulfite as recommended by Van Soest et al. (1991). All fiber values were corrected for residual ash. The fermentation gases were analyzed daily for concentrations of methane with a gas chromatograph (model 5890 Series II, Hewlett Packard, Avondale, PA, USA) equipped with an FID and WLD detector using a 2.34 m × 2.3 mm column (80/100 mesh, Porapak Q, Fluka Chemie AG, Buchs, Switzerland). For more details see Soliva and Hess (2007).

### Calculations and statistical analysis

The apparent *in vitro* nutrient degradability was estimated from the disappearance of nutrients from the nylon bags during 48 h of incubation. Incubation fluid N turnover was calculated from the N disappearance rates and the daily amounts of ammonia produced. Four N fractions were computed including (i) N recovered as ammonia, (ii) N present in compounds apparently not degraded (representing the rumen-undegraded protein), (iii) RDP calculated as proportion of feed CP not recovered in the fermentation residues (i.e., apparently degraded CP), and (iv) N from compounds apparently degraded (i.e., disappeared from the fermentation residue) but not recovered as ammonia N. The latter fraction was calculated as total feed-N (i.e., inclusive of urea) degraded minus ammonia-N. Assuming that (at low CP supply) RDP is limiting OM degradation, the amount of RDP found with a distinct amount of OM degraded (i.e., ratio between these two variables) was considered to be equivalent to the RDP amount required to achieve this level of degradation.

Data of the main experiment was subjected to regression analysis in SigmaPlot™ (version 12 for Windows, Systat Software GmbH, Germany). Maximizing adjusted *R*^2^ was the criterion used to select either linear or non-linear regression models while unadjusted *R*^2^-values are presented for the selected models in the figures. The equations are characterized in addition with standard errors (SE) and significance for individual coefficients and the overall equation. Significance of difference between the regression coefficients among the three fiber sources was determined using the Proc Reg Data Model described by the Institute for Digital Research and Education (2015) using SAS (version 9.2 for Windows, SAS Institute, Cary, USA). Thereby, all regression coefficients of first order term were compared as a whole to test the null hypothesis: fiber source 1 = fiber source 2 = fiber source 3. A 95% confidence interval was chosen. In case there was a statistical significance, this meant that the null hypothesis for this relationship could be rejected, i.e., the respective trait investigated in the fiber sources (y) did not respond in the same way to the effect (x). In order to further characterize the fiber sources, data averaged across all urea treatments was also evaluated by analysis of variance (procedure GLM) with SAS, where fiber sources, i.e., brachiaria hay, apple pomace, and beet pulp, were considered as the only source of variation. In this context, the N-related variables were not analyzed, as they were directly dependent on urea supplementation. Means were compared with Tukey's method. For all statistical evaluations the level of statistical significance was set to *P* < 0.05.

## Results

The three test feeds differed in fiber properties as intended, with a high fiber concentration found in the brachiaria hay, a high lignification in the apple pomace and medium values for fiber and lignification in the beet pulp (Table 1). The hemicellulose-to-cellulose ratios were 0.77, 0.88, and 1.25 for brachiaria hay, apple pomace and beet pulp, respectively. Beet pulp had the highest CP concentration, followed by brachiaria hay and apple pomace. On average across all urea concentrations, incubation fluid pH was lowest (*P* < 0.05) with beet pulp. Urea supplementation did not affect incubation fluid pH (*P* = 0.92; data not shown). Apple pomace resulted in the lowest bacteria and the highest protozoa counts (*P* < 0.05). Total SCFA were higher with beet pulp than with apple pomace (*P* = 0.001). Fiber source affected proportions of all individual SCFA except propionate, with apple pomace being different (lower in acetate and higher in most other SCFA proportions) from the two other fiber sources. The OM, NDF, and ADF from apple pomace had the lowest (*P* < 0.05) apparent degradability followed by the brachiaria hay. Brachiaria hay did not significantly differ in OM degradability from apple pomace, and, in case of OM and ADF degradability, from beet pulp. The RDP amount required per unit of OM apparently degraded was highest (*P* < 0.05) with the brachiaria hay with values being similar with the two other feeds. The ratio of RDP-to-fermented NDF was higher with apple pomace and brachiaria hay than with beet pulp, while RDP-to-fermented ADF did not significantly differ between feeds. Absolute methane formation showed a trend to be highest (*P* = 0.055) with beet pulp. Methane per unit of OM degraded did not differ among feeds. When methane formation was related to units of NDF degraded, methane was highest with apple pomace, lowest with brachiaria hay (*P* < 0.05), and intermediate with beet pulp.

**Figure 1:**
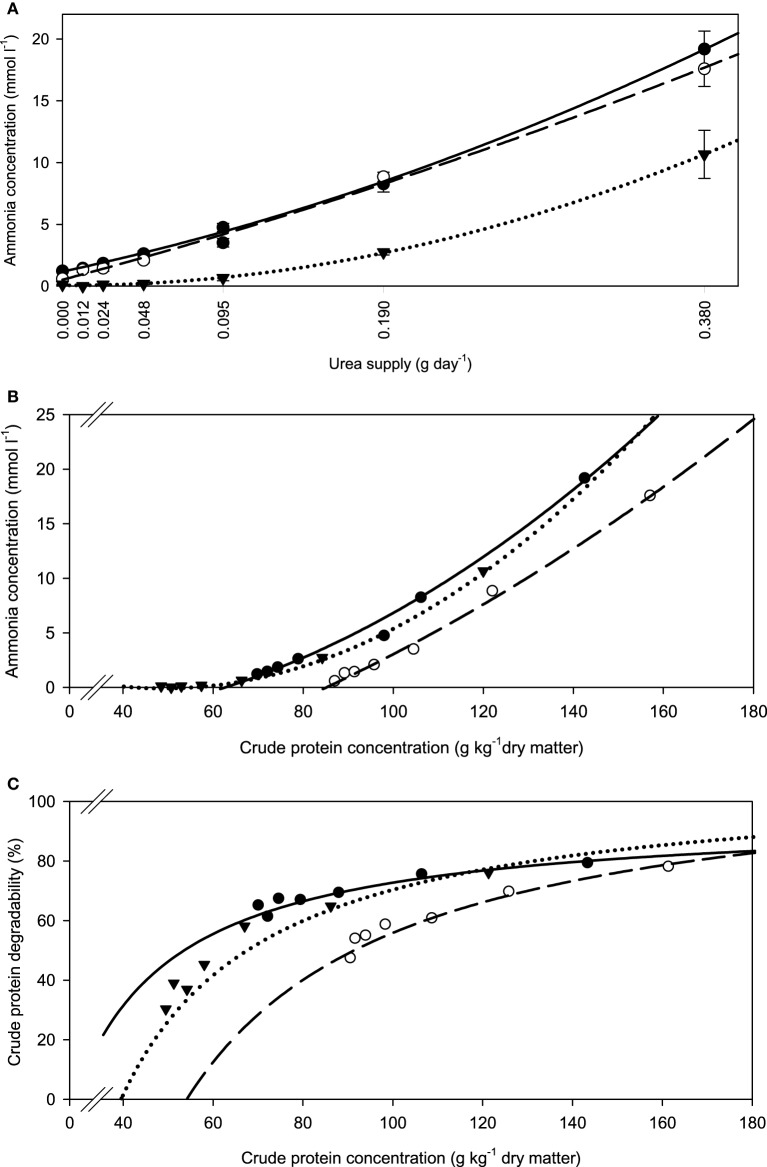
**Relationship between (A) incubation fluid ammonia concentration and urea supply, (B) incubation fluid ammonia concentration and dietary crude protein concentration, and (C) crude protein degradability and dietary crude protein concentration**. 
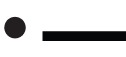
 brachiaria hay, for **(A)** *y* = 1.18 (±0.134; *P* < 0.001) + 29.4 (±2.47; *P* < 0.001) x + 47.1 (±6.35; *P* = 0.0018) x^2^, *R*^2^ = 1.00, *SE* = 0.217, *P* < 0.001, **(B)** *y* = 4.58 (±4.56; *P* = 0.372) −0.191 (±0.0922; *P* = 0.107) x +0.00206 (±0.00038; *P* = 0.0092) x^2^, *R*^2^ = 0.994, *SE* = 0.599, *P* < 0.001, **(C)** *y* = 21.4 (±16.9; *P* = 0.2733) + 0.793 (±0.340; *P* = 0.0799) x −0.00270 (±0.00159; *P* = 0.1651) x^2^, *R*^2^ = 0.928, *SE* = 2.03, *P* = 0.055; 
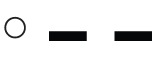
 beet pulp, for **(A)** *y* = 0.530 (±0.307; *P* = 0.1595) + 36.5 (±5.66 x; *P* = 0.0030) + 22.9 (±14.6; *P* = 0.1911) x^2^, *R*^2^ = 0.996, *SE* = 0.498, *P* < 0.001, **(B)** y = −11.5 (±6.06; *P* = 0.130) + 0.0801 (±0.1044; *P* = 0.486) x + 0.000676 (±0.00043; *P* = 0.190) x^2^, *R*^2^ = 0.996, *SE* = 0.498, *P* < 0.001, **(C)** *y* = −43.1 (±32.9; *P* = 0.261) + 1.42 (±0.551; *P* = 0.0614) x − 0.00421 (±0.00223; *P* = 0.132) x^2^, *R*^2^ = 0.956, *SE* = 2.67, *P* = 0.002; 
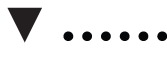
 apple pomace, for **(A)** *y* = 0.066 (±0.036; *P* = 0.145) − 0.253 (±0.667; *P* = 0.724) x + 74.2 (±1.73; *P* < 0.001) x^2^, *R*^2^ = 1.00, *SE* = 0.0592, *P* < 0.001, **(B)** y = 5.04 (±0.307; *P* < 0.001) − 0.204 (±0.0081; *P* < 0.001) x + 0.00209 (±0.000048; *P* < 0.001) x^2^, *R*^2^ = 1.00, *SE* = 0.0585, *P* < 0.001, **(C)** *y* = −57.6 (±21.0; *P* = 0.0514) + 2.36 (±0.548; *P* = 0.0126) x − 0.0104 (±0.0032; *P* = 0.0319) x^2^, *R*^2^ = 0.963, *SE* = 3.92, *P* = 0.0013. The significance of the difference of response across fiber sources was *P* = 0.001, *P* < 0.001, and *P* = 0.167 for **(A–C)**, respectively.

The fiber sources responded differently (*P* < 0.001) in incubation fluid ammonia concentration to increasing urea supplementation. The slope of development appeared similar in all three feeds, but with apple pomace this seemed to happen at a generally lower level at any urea supplementation level (Figure 1A). These differences were less obvious when relating incubation fluid ammonia concentration to dietary CP (Figure 1B), but the response still differed (*P* < 0.001) across fiber sources. The percentage of dietary CP apparently degraded increased with increasing urea supplementation and eventually approached a plateau at around 80% CP degradability (Figure 1C).

**Figure 2:**
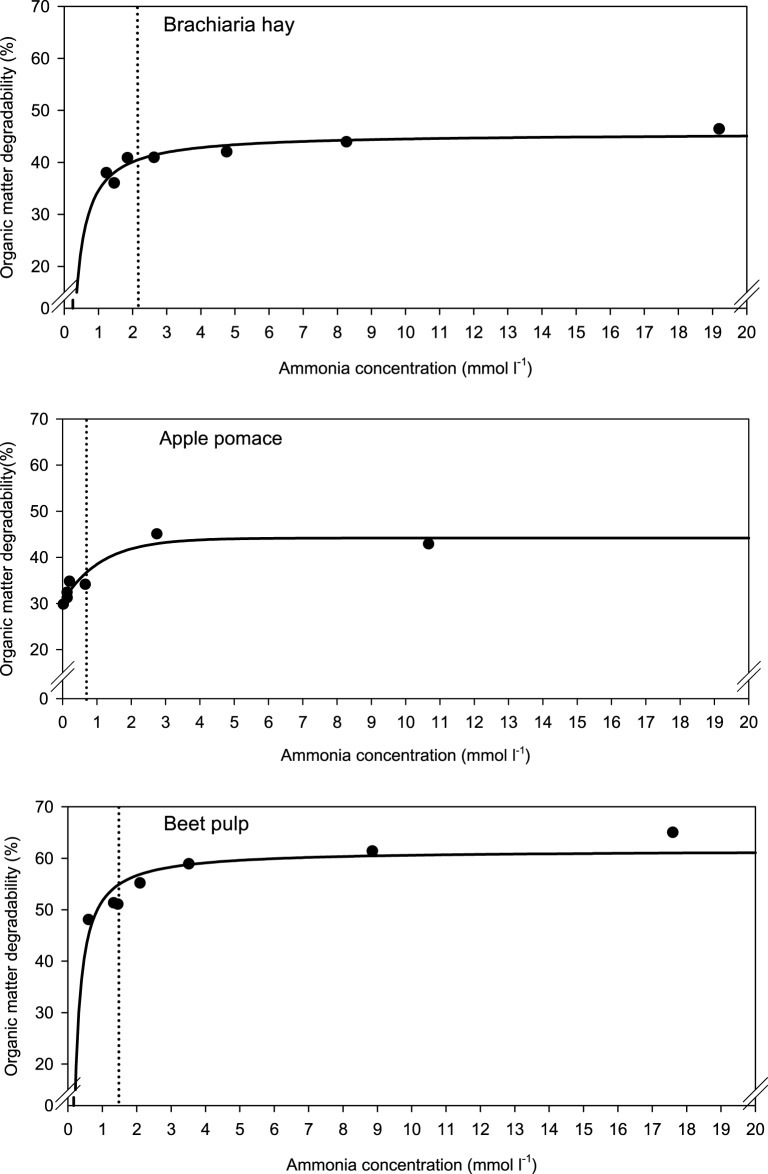
**Relationship between apparent organic matter degradability and incubation fluid ammonia concentration**. The intersection between the vertical dotted line and the x-axis represents the ammonia concentration at which 95% of the calculated maximal degradability was achieved. For brachiaria hay: *y* = 45.6 (±1.02; *P* < 0.001) − 11.0 (±2.11; *P* = 0.0034) x^−1^, *R*^2^ = 0.846, *SE* = 1.50, *P* = 0.0034; for apple pomace: *y* = 30.4 (±1.46; *P* < 0.001) + 13.8 (±2.12; *P* = 0.0029) [1−0.411 (±0.202; *P* = 0.1118)^x^], *R*^2^ = 0.915, *SE* = 2.11, *P* = 0.0073; for beet pulp: *y* = 61.6 (±1.82; *P* < 0.001) − 9.86 (±2.35; *P* = 0.0085) x^−1^, *R*^2^ = 0.787, *SE* = 3.18, *P* < 0.001. The significance of the difference of response across fiber sources was *P* = 0.159.

**Figure 3:**
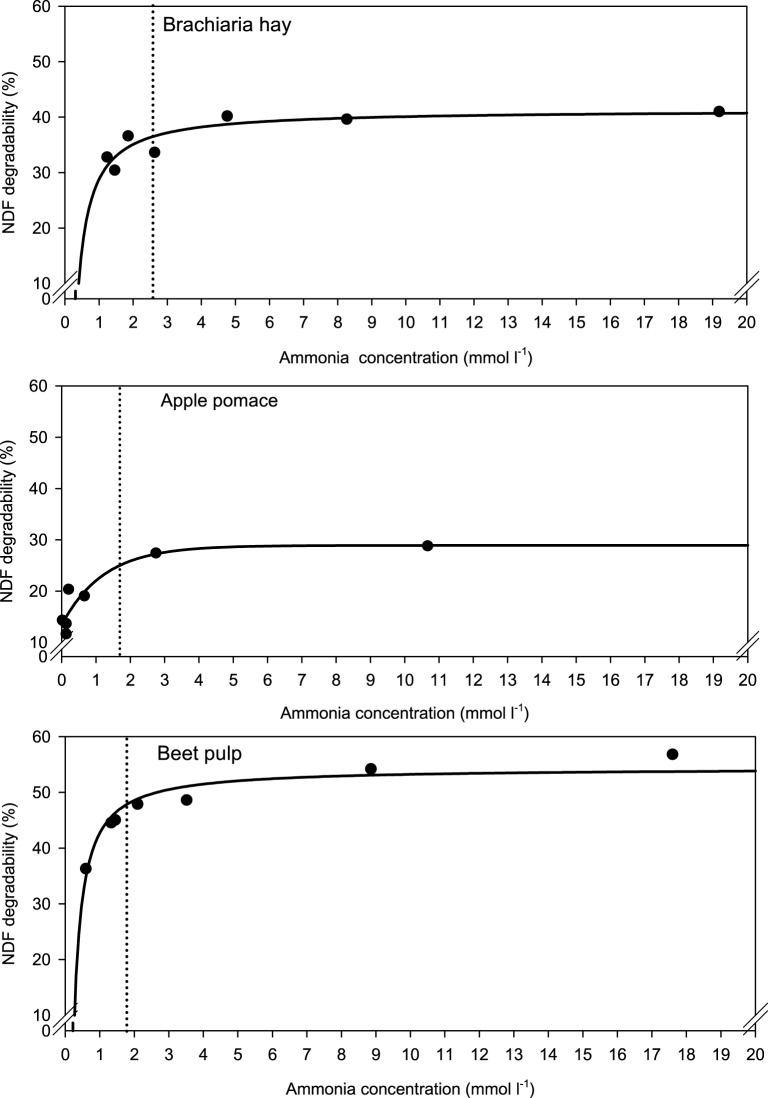
**Relationship between apparent neutral detergent fiber (NDF) degradability and incubation fluid ammonia concentration**. The intersection between the vertical dotted line and the x-axis represents the ammonia concentration at which 95% of the calculated maximal degradability was achieved. For brachiaria hay: *y* = 41.4 (±1.46; *P* < 0.001) − 12.5 (±3.02; *P* = 0.0090) x^−1^, *R*^2^ = 0.775, *SE* = 2.15, *P* = 0.0090; for apple pomace: *y* = 13.4 (±2.00; *P* = 0.0025) + 15.5 (±3.02; *P* = 0.0069) [1 − 0.444 (±0.251; *P* = 0.1522)^*x*^], *R*^2^ = 0.870, *SE* = 2.98, *P* = 0.017; for beet pulp: *y* = 54.4 (±1.22; *P* < 0.001) −11.7 (±1.57; *P* < 0.001) x^−1^, *R*^2^ = 0.917, *SE* = 2.13, *P* < 0.001. The significance of the difference of response across fiber sources was *P* = 0.099.

**Figure 4:**
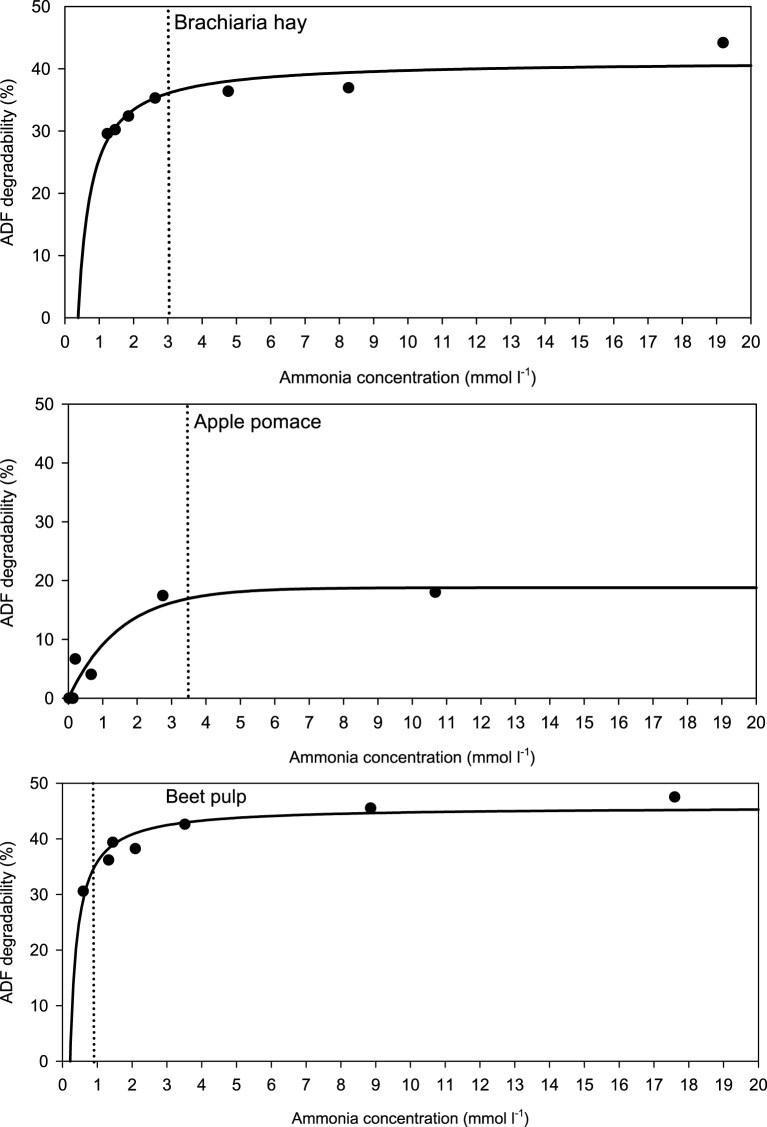
**Relationship between apparent acid detergent fiber (ADF) degradability and incubation fluid ammonia concentration**. The intersection between the vertical dotted line and the x-axis represents the ammonia concentration at which 95% of the calculated maximal degradability was achieved. For brachiaria hay: *y* = 41.3 (±1.48; *P* < 0.001) − 15.8 (±3.06; *P* = 0.0036) x^−1^, *R*^2^ = 0.841, *SE* = 2.18, *P* = 0.0036; for apple pomace: *y* = 18.8 (±2.49; *P* < 0.001) [1 − 0.514 (±0.141; *P* = 0.0147)^*x*^], *R*^2^ = 0.911, *SE* = 2.61, *P* < 0.001; for beet pulp: *y* = 45.8 (±1017; *P* < 0.001) − 9.91 (±1.51; *P* = 0.0012) x^−1^, *R*^2^ = 0.896, *SE* = 2.04, *P* = 0.0012. The significance of the difference of response across fiber sources was *P* = 0.064.

When relating apparently degraded OM to incubation fluid ammonia, curves with mostly pronounced plateaus were found irrespective of the type of regression equation used (Figure 2) and the response did not differ across fiber sources. This was different for degraded NDF and ADF in relation to incubation fluid ammonia with trends (*P* = 0.099 and *P* = 0.064, respectively) to differ across the three feeds (Figures 3, 4). Clear but largely differing plateaus in NDF and ADF degradability developed with increasing amounts of urea as had also been the case for OM degradability. The ammonia concentrations where 95% of the maximum was reached ranged between 0.75 and 2.19, 1.73, and 2.54, as well as 0.89 and 3.48 mmol l^−1^ for OM, NDF, and ADF degradability.

**Figure 5:**
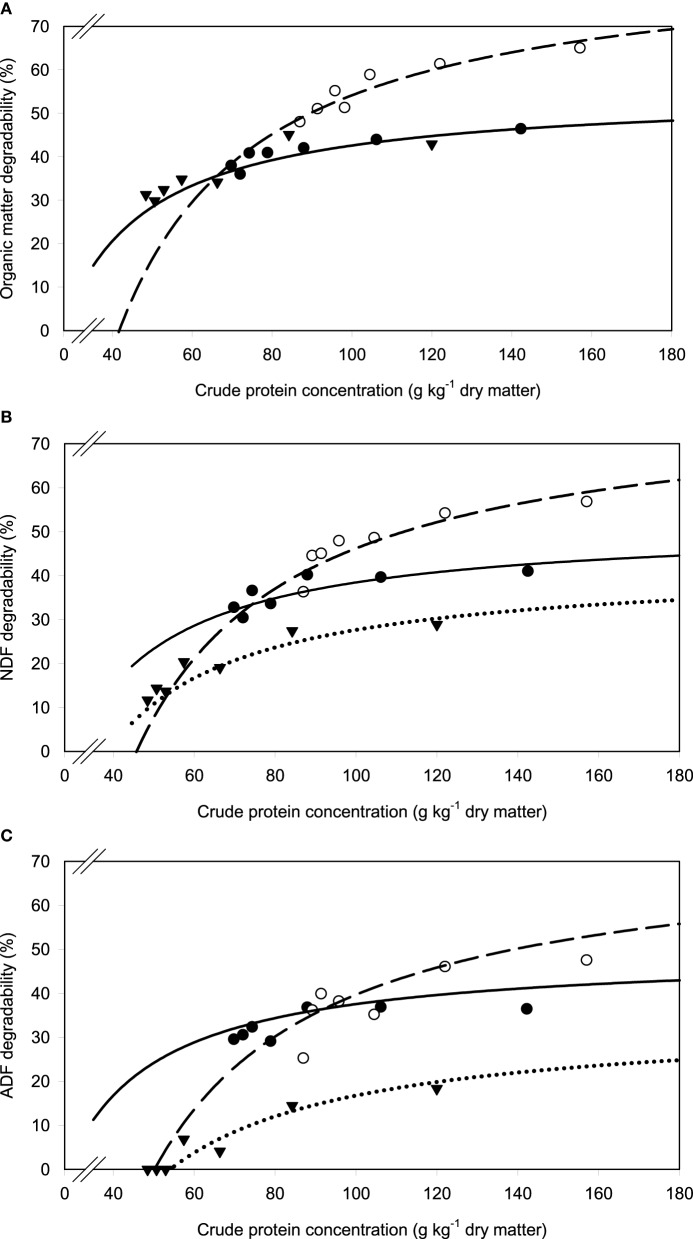
**Relationship between apparent degradability of organic matter (A), neutral detergent fiber (NDF) (B) and acid detergent fiber (ADF) (C) and dietary crude protein concentration**. 
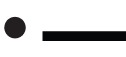
 brachiaria hay, for **(A)** *y* = 55.3 (±2.70; *P* < 0.001) − 1203 (±225; *P* = 0.0031) x^−1^, *R*^2^ = 0.851, *SE* = 1.47, *P* = 0.003, **(B)** *y* = 51.4 (±4.6; *P* < 0.001) − 1283 (±385; *P* = 0.0208) x^−1^, *R*^2^ = 0.689, *SE* = 2.52, *P* = 0.0208, **(C)** *y* = 45.6 (±4.20; *P* < 0.001) − 1064 (±350; *P* = 0.0287) x^−1^, *R*^2^ = 0.649, *SE* = 2.29, *P* = 0.029; 
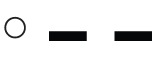
 beet pulp, for **(A)** *y* = 87.4 (±5.06; *P* < 0.001) − 3289 (±519; *P* = 0.0015) x^−1^, *R*^2^ = 0.889, *SE* = 2.26, *P* = 0.002, **(B)** *y* = 79.6 (±6.72; *P* < 0.001) − 3272 (±679; *P* = 0.0048) x^−1^, *R*^2^ = 0.823, *SE* = 3.11, *P* = 0.005, **(C)** *y* = 69.3 (±10.6; *P* = 0.0012) − 3178 (±1068; *P* = 0.0310) x^−1^, *R*^2^ = 0.639, *SE* = 4.90, *P* = 0.031; 
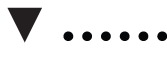
 apple pomace, for **(A)** *y* = 54.9 (±3.93; *P* < 0.001) − 1191 (±238; *P* = 0.0041) x^−1^, *R*^2^ = 0.834, *SE* = 2.62, *P* = 0.004, **(B)** *y* = 42.2 (±3.16; *P* < 0.001) − 1430 (±191; *P* < 0.001) x^−1^, *R*^2^ = 0.918, *SE* = 2.12, *P* < 0.001, **(C)** *y* = 31.6 (±3.53; *P* < 0.001) − 1555 (±214; *P* < 0.001) x^−1^, *R*^2^ = 0.917, *SE* = 2.37, *P* < 0.001. The significance of the difference of response across fiber sources was *P* = 0.074, 0.064, and 0.033 for organic matter **(A)**, neutral detergent fiber **(B)**, and acid detergent fiber **(C)**, respectively.

A relatively clear plateau in apparent OM, NDF, and ADF degradability was found with brachiaria hay and apple pomace from dietary CP concentrations of about 80 g kg^−1^ DM onwards, whereas no such plateau was found with beet pulp (Figure 5). In all relationships, there was a trend (*P* < 0.10) for a difference in response across fiber sources.

**Figure 6:**
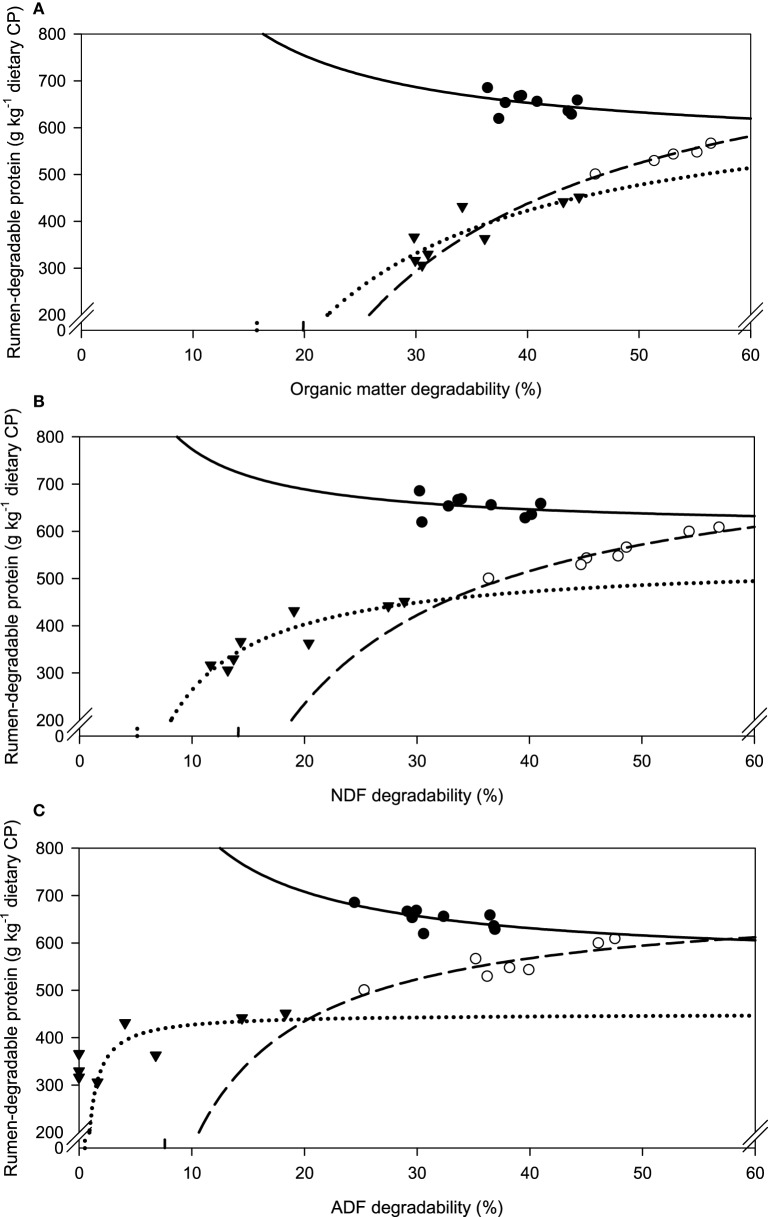
**Relationship between apparent degradability of organic matter (A), neutral detergent fiber (ADF) (B) and acid detergent fiber (ADF) (C) and rumen-degradable protein proportion in dietary crude protein**. 
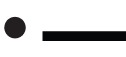
 brachiaria hay, for **(A)** *y* = 552 (±101; *P* < 0.001) + 40.3 × 10^2^ (±40.5 × 10^2^; *P* = 0.3527) x^−1^, *R*^2^ = 0.242, *SE* = 21.0, *P* = 0.3527, **(B)** *y* = 604 (±66.3; *P* < 0.001) + 17.0 × 10^2^ (±23.03 × 10^2^; *P* = 0.4849) x^−1^, *R*^2^ = 0.0721, *SE* = 21.6, *P* = 0.485, **(C)** *y* = 555 (±43.2; *P* < 0.001) + 30.7 × 10^2^ (±13.4 × 10^2^; *P* = 0.0554) x^−1^, *R*^2^ = 0.429, *SE* = 16.7, *P* = 0.055. 
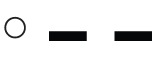
 beet pulp. for **(A)** *y* = 871 (±19.3; *P* < 0.001) − 17.3 × 10^3^ (±10.6 × 10^2^; *P* = 0.0140) x^−1^, *R*^2^ = 0.982, *SE* = 5.68, *P* < 0.001, **(B)** *y* = 797 (±34.8; *P* < 0.001) −112 × 10^2^ (±16.1 × 10^2^; *P* < 0.001) x^−1^, *R*^2^ = 0.9066, *SE* = 12.8, *P* < 0.001, **(C)** *y* = 701 (±39.8; *P* < 0.001) −53.2 × 10^2^ (±14.4 × 10^2^; *P* = 0.0140) x^−1^, *R*^2^ = 0.732, *SE* = 21.7, *P* = 0.014. 
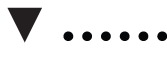
 apple pomace, for **(A)** *y* = 697 (±83.6; *P* < 0.001) −11.0 × 10^3^ (±28.3 × 10^2^; *P* = 0.0082) x^−1^, *R*^2^ = 0.715, *SE* = 33.8, *P* = 0.0082, **(B)** *y* = 541 (±33.0; *P* < 0.001) −27.7 × 10^2^ (±5.29 × 10^2^; *P* = 0.0020) x^−1^, *R*^2^ = 0.820, *SE* = 26.8, *P* = 0.002, **(C)** *y* = 451 (±25.9; *P* < 0.001) −231 (±85.3; *P* = 0.0735) x^−1^, *R*^2^ = 0.709, *SE* = 38.9, *P* = 0.074. The significance of the difference of response across fiber sources was *P* = 0.018, 0.018, and 0.008 for organic matter **(A)**, neutral detergent fiber **(B)**, and acid detergent fiber **(C)**, respectively.

**Figure 7:**
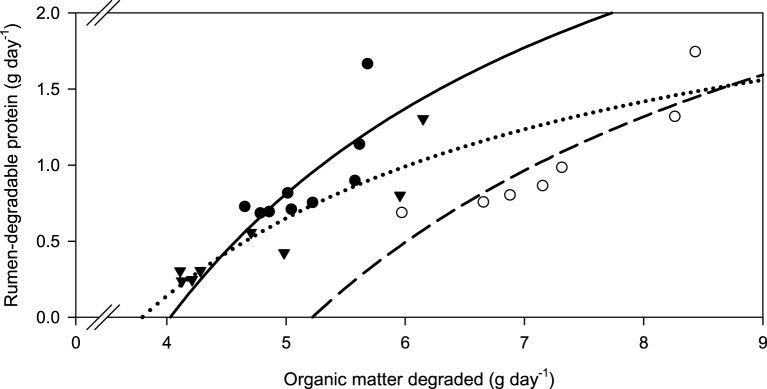
**Relationship between the amounts of organic matter degraded and rumen-degradable protein**. 
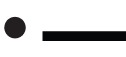
 brachiaria hay, *y* = 4.18 (±1.09; *P* = 0.0064) − 16.8 (±5.57; *P* = 0.0194) x^−1^, *R*^2^ = 0.566, *SE* = 2.23, *P* = 0.019; 
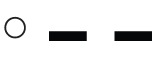
 beet pulp, *y* = 3.80 (±0.666; *P* = 0.0023) − 19.8 (±4.73; *P* = 0.0086) x^−1^, *R*^2^ = 0.778, *SE* = 0.196, *P* = 0.0086; 
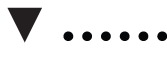
 apple pomace, *y* = 2.70 (±0.411; *P* < 0.001) − 10.2 (±1.91; *P* = 0.0017) x^−1^, *R*^2^ = 0.827, *SE* = 0.166, *P* = 0.017. The significance of the difference of response across fiber sources was *P* < 0.001.

Along with increasing OM, NDF, and ADF degradability, the amount of rumen-degradable protein (in dietary CP) was increased for apple pomace and beet pulp (Figure 6). In the brachiaria hay no such relationship was apparent. Accordingly, the responses differed (*P* < 0.05) across fiber sources for all groups of nutrients fermented. There was a clear dependence of the amount of RDP required for increasing amounts of OM fermented, but the response across fiber sources differed (*P* < 0.001, Figure 7).

**Figure 8:**
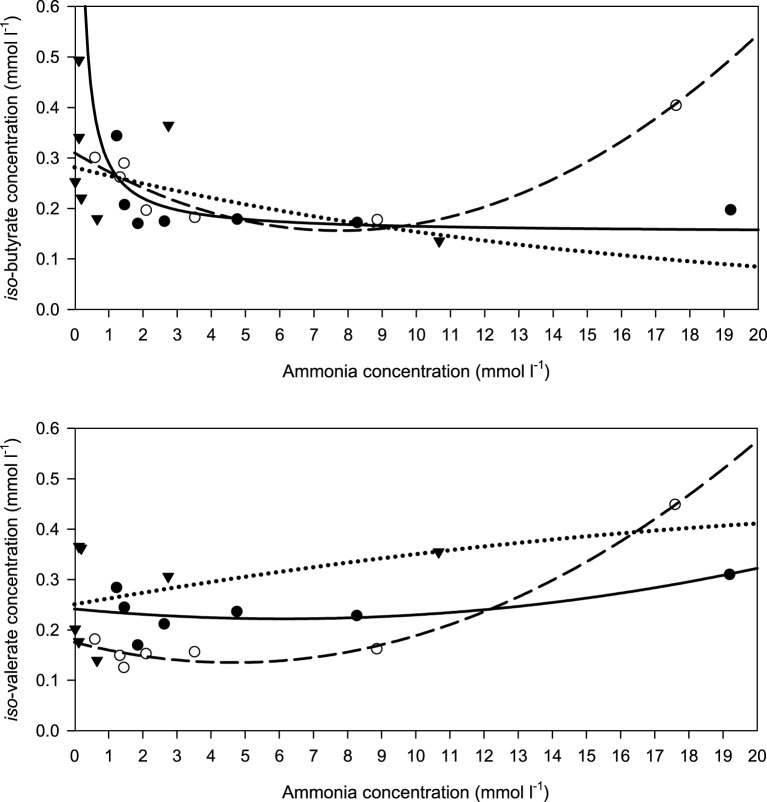
**Relationship between the concentrations of incubation fluid *iso*-butyrate and *iso*-valerate and ammonia concentration**. 
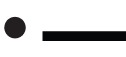
 brachiaria hay, for *iso*-butyrate: *y* = 0.151 (±0.0351; *P* = 0.0077) + 0.139 (±0.0728; *P* = 0.1144) x^−1^, *R*^2^ = 0.422, *SE* = 0.0518, *P* = 0.114, for *iso*-valerate: *y* = 0.242 (±0.034; *P* = 0.00021) − 0.00641 (±0.01193; *P* = 0.6213) x + 0.000520 (±0.00057; *P* = 0.4147) x^2^, *R*^2^ = 0.460, *SE* = 0.0414, *P* = 0.292, 
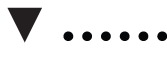
 apple pomace, for *iso*-butyrate: *y* = 0.282 (±0.0483; *P* = 0.0021) exp[0.0602 (±0.0514; *P* = 0.2939) x], *R*^2^ = 0.303, *SE* = 0.206, *P* = 0.200, for *iso*-valerate: *y* = 0.251 (±0.0551; *P* = 0.0104) + 0.0119 (±0.0622; *P* = 0.8577) x − 0.000194 (±0.00570; *P* = 0.9746) x^2^, *R*^2^ = 0.157, *SE* = 0.109, *P* = 0.7110, 
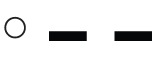
 beet pulp, for *iso*-butyrate: *y* = 0.310 (±0.0218; *P* < 0.001) − 0.0398 (±0.0088; *P* = 0.0106) x + 0.00259 (±0.00052; *P* = 0057) x^2^, *R*^2^ = 0.909, *SE* = 0.302, *P* = 0.0082, for *iso*-valerate: *y* = 0.176 (±0.0143; *P* < 0.001) − 0.0173 (±0.0058; *P* = 0.0401) x + 0.00186 (±0.00031; *P* = 0.0040) x^2^, *R*^2^ = 0.979, *SE* = 0.0198, *P* < 0.001. The significance of the difference of response across fiber sources was *P* = 0.024 and *P* = 0.014 for *iso*-butyrate and *iso*-valerate, respectively.

When relating the concentration of the two branched SCFA to incubation fluid ammonia concentration, there was a curvilinear response with beet pulp and, less clearly, with brachiaria hay where elevated levels occurred at low (especially with *iso*-butyrate) and at high ammonia concentrations (Figure 8). The responses differed (*P* < 0.05) across fiber sources in both SCFA.

## Discussion

### Composition of the experimental feeds

The differences in fiber concentration and degree of lignification among the three feeds were realized as intended. This affected the apparent degradability of OM, NDF, and ADF across all urea treatments with the expected increases from apple pomace to brachiaria hay to beet pulp. The two extremes in this respect, which were represented by apple pomace and beet pulp, were still in the range of the known variation in OM and fiber digestibility for fibrous feeds (DLG, 1997). The CP concentrations of the test batches of apple pomace and beet pulp used were slightly lower than those listed by DLG (1997) and NRC (2001). The apple pomace contained less NDF and ADF at quite similar ADL concentration compared to average values given in NRC (2001). In beet pulp, concentrations of NDF and ADF were as expected, but ADL concentrations was unexpectedly high (77 vs. 16 g kg^−1^ DM as given in NRC, 2001). The extensive fermentation of the beet pulp explains the comparably lower incubation fluid pH and the high SCFA and methane formation. The high protozoa count and the low bacteria count found with apple pomace in comparison to the other feeds might have resulted from its typically substantial residual sugar concentration (Hindrichsen et al., 2004). This fiber source also led to a deviating SCFA profile with a shift from acetate to butyrate (and valerate; inclusive of the branched forms of these SCFA) as is expected from the action of sugar-fermenting microbes. The *Brachiaria* species used (*B. brizantha*) was richer in CP and lower in NDF than other *Brachiaria* species (e.g., *B. humidicola*, also called *B. dictyoneura*; with CP concentrations of <40 g kg^−1^; Hess et al., 2003; Tiemann et al., 2008a), while ADF and ADL concentrations were comparable to those of other *Brachiaria* species.

### Potential limitations in organic matter and fiber degradation

The results from the first preliminary assessment demonstrate that the known rumen microbial growth factors were present in the rumen fluid in sufficient amounts to cover the microbial needs for the entire 10 days of incubation. Russell et al. (1992) implicated that the only N source required by microbes to ferment structural carbohydrates is ammonia. However, stimulation of ruminal microbiota by preformed amino acids (AA), especially branched-chain AA and SCFA (Gorosito et al., 1985), has been shown. This is especially true for the cellulolytics, as was shown for three main species, namely *Fibrobacter succinogenes, Ruminococcus albus*, and *R. flavefaciens*, by Atasoglu et al. (2001). These branched carbon sources can be provided via rumen-degradable protein, peptides or AA (e.g., Newbold, 1999; Ranilla et al., 2001). Nevertheless, no pre-formed AA or other such stimulants were supplemented in the present main experiment. One reason was that urea would be the least expensive supplementation strategy available to economically disadvantaged tropical farmers and is, therefore, more likely adopted, e.g., in the form of urea-molasses licking blocks or urea-treated straw and hay. Besides this, effects of urea and preformed AA would have inseparably contributed to RDP supply, and it seems that a deficiency of AA, if any, would uncouple carbohydrate degradation from microbial protein synthesis which then would be noticeably impaired (reviewed by Owens and Bergen, 1983). Griswold et al. (2003) noted that a lack of urea in the infusate also decreased proteolysis of the feed which would indicate that decreasing ammonia concentration could stress the competition for preformed amino-N vs. ammonia-N in the RDP available. Still, the present result that there is an increase in the concentration of the branched SCFA under the condition of very low ammonia concentration suggests the opposite and indicates that there was no need for supplementing preformed AA under the conditions investigated.

### Responses in nutrient degradability to extra urea and to increasing incubation fluid ammonia concentration

Urea was used to increase the dietary proportion of RDP. The three feeds provided RDP themselves and this at different levels. Therefore, the ammonia concentrations without extra urea, i.e., the starting points, were different with 1.2, 0.1, and 0.6 mmol l^−1^ for brachiaria hay, apple pomace and beet pulp, respectively. However, any of these concentrations were low as is characteristic for RDP-poor feeds. The regression analysis demonstrated the presence of clear responses in apparent OM and fiber degradability to increasing incubation fluid ammonia concentrations. Due to their particularly high demand for ammonia, the fibrolytic microbes obtain a specific advantage with increasing ammonia concentrations because the micro-environment is then better equilibrated with ammonia across the entire rumen (Owens and Bergen, 1983). As a limitation, it has to be stated that, compared to Rusitec, *in vivo* there might be compartmentalization preventing the establishment of a complete equilibration. This was shown by Storm et al. (2012) for the SCFA. In an earlier study (Tiemann et al., 2008a), supplementing 0.167 g urea day^−1^ to 15 g hay day^−1^ of *B. dictyoneura* resulted in an increase in incubation fluid ammonia from 0.6 to 4.4 mmol l^−1^ and in apparent NDF degradability from 24 to 36% in Rusitec. This is comparable with the responses found in the present study with *B. brizantha*. Also in the study by Riemeier et al. (2004), ruminal OM degradability in cows was at a moderate level (66%) at a very low ruminal ammonia concentration of 1.1 mmol l^−1^, and OM apparently degraded increased to 70% at a ruminal ammonia concentration of about 10 mmol l^−1^, but even slightly declined when ammonia concentration increased to 20 mmol l^−1^. Intake of digestible OM was found to increase with supplementary RDP when feeding tallgrass-prairie hay containing <50 g kg^−1^ CP, starting at ruminal ammonia concentration as low as 0.3 mmol ammonia l^−1^ (Olson et al., 1999). Extra urea also increased OM digestibility in sheep in a curvilinear, i.e., more than proportionate manner, starting at a ruminal ammonia concentration of 2.7 mmol l^−1^ (Balcells et al., 1993). Increasing RDP levels in a low-quality grass hay diet of beef steers by the supplementation with sodium caseinate from 0 to 0.64 kg day^−1^ increased ruminal ammonia concentration from 0.62 to 11.22 mmol l^−1^ and concomitantly elicited a positive quadratic response in NDF degradability (Klevesahl et al., 2003). In contrast, Sawyer et al. (2012) did not find an effect on NDF digestibility when supplementing a cattle diet with urea of 0, 40, 80, and 160 g d^−1^ while exhibiting a quadratic response in ruminal ammonia concentration. In the study of Mathis et al. (2000), the response of OM digestibility in steers to RDP (casein) clearly differed among several low-CP forages. Overall, these rather variable findings suggest that extra RDP enhances ruminal ammonia concentration, sometimes in a linear fashion, and sometimes non-linearly. In addition this often, but not always, increases ruminal OM and fiber degradability.

### Requirements for rumen-degradable protein with different feeds

There are various individual ways for describing RDP requirements of ruminal microbes in order to enable them to ferment OM and fiber efficiently. The most direct trait builds on rumen fluid ammonia concentration. Recommendations for minimum ammonia concentration range from slightly above 1 (Satter and Slyter, 1974; Marini and Van Amburgh, 2003) up to 16 mmol l^−1^ (Mehrez et al., 1977) with other recommendations ranging in between (reviewed by Balcells et al., 1993; Schwab et al., 2005). In the present study, when either apparent OM or fiber fermentation approached a plateau, the ammonia concentration was in the lower range of these studies accounting for either 0.75–2.19 or 1.73–2.54 mmol l^−1^, respectively (depending on the regression equation). Thereby, the threshold for the ammonia concentration required largely differed among test feeds. It was reached first with apple pomace then with beet pulp and finally with the brachiaria hay. Consistent with this, Calsamiglia et al. (2010) stated in their review that the use of ammonia-N as the sole criterion for determining minimum concentration of N for optimal microbial growth should be challenged.

Assessing ruminal ammonia concentration is impractical on farm. Thus, dietary indicators are preferable. The concept of a minimum dietary CP concentration was often applied in the past, with 130–160 g CP kg^−1^ being considered to supply sufficient amounts of RDP (Schwab et al., 2005). Huhtanen and Hristov (2009) even concluded from a meta-analysis that the N fractions currently recommended to be incorporated in feeding systems (RDP and RUP, intestinal digestion, etc.) do not seem to improve our ability to optimize N utilization, and only the CP concentration of the diet appears to be closely related to the efficiency of N utilization. Still minimum requirements for RDP in order to maximize fiber degradation were evaluated by regression analysis of various studies and compiled in the NRC (2001) recommendations. These requirements amount to about 100 g RDP kg^−1^ (assuming constant DM intake and non-rumen degradable protein levels). Schwab et al. (2005) compiled data indicating that requirements are actually lower with 50–80 g RDP kg^−1^ DM. In the present study, the relatively clear plateau in apparent OM and NDF degradability found with brachiaria hay and apple pomace was starting at a dietary CP level of about 80 g kg^−1^ DM. The CP was widely equivalent to RDP in this context because most of it consisted of supplemented urea. Considering that still part of the CP was undegradable because total CP also included in the test feeds, the present results fall in the range specified by Schwab et al. (2005). By contrast, the situation with the best fermentable fiber source, beet pulp, was different from that found with brachiaria hay and apple pomace, and the results indicated requirements close to the apparently higher amount of RDP recommended by NRC (2001). The increasing proportion of OM degradability with increasing dietary CP supplementation (going up to 180 g CP kg^−1^) eventually even resulted in the lack of the development of a clear plateau with beet pulp. Schwab et al. (2005) stated that the need for supplemental RDP might be related more to forage CP concentration than to ruminal ammonia concentration. This seems to be true for the beet pulp in the present study as a plateau in apparent OM and NDF degradability was reached even when incubation fluid ammonia concentration further increased.

New concepts of feeding recommendation build on RDP: apparently degraded OM ratios (Bach et al., 2005; Schwab et al., 2005). Others calculate ruminal N balance giving either percentages (NRC, 2001) or the deviation from stated ruminal N MJ^−1^ (metabolizable) energy (DLG, 1997; Riemeier et al., 2004). The latter approaches conceptually do not differ much from the RDP: apparently degraded OM ratio principle. In the present study, the requirements for RDP per unit of OM apparently degraded were similar with apple pomace and beet pulp, but were much higher for the brachiaria hay. Actually, it appeared from the regressions that these requirements did not change at all in the range investigated when rumen degradability of CP was concerned and thus absolute RDP requirements almost linearly increased with the amount of OM fermented. Based on the observations with low-CP tropical diets, the opposite had been expected. Differences in apparent OM and fiber degradability without urea supplementation do not yield an explanation because the brachiaria hay ranged in the middle of the two other feeds in this respect. The observation of a higher RDP: apparently degraded OM ratio with brachiaria hay is, however, consistent with the finding of a particularly high incubation fluid ammonia concentration which was obviously required to approach maximum apparent OM and fiber degradability. In the study by Mathis et al. (2000), most RDP was required to digest OM when using bermuda grass, whereas less RDP was needed with bromegrass and forage sorghum. Therefore, it still remains to be investigated whether this phenomenon is typical for at least some tropical grasses or whether feeds like apple pomace and beet pulp require smaller amounts of RDP in order to ferment OM and fiber. Explanations for these clear, although unexpected, findings might be sought in differences in the microenvironment, in addition to variations caused in N recycling as presumed by Mathis et al. (2000). Accordingly, Odle and Schaefer (1987) presumed that the optimum ammonia concentration is affected by chemical or structural characteristics. Definitely, the requirements for RDP for fermenting OM and fiber differed between the fibrous feeds and there was no such phenomenon of a simple overall threshold level to be recommended as it would be desirable and is sometimes practiced.

## Conclusion

The hypothesis that the amount of RDP required to ferment OM and fiber differs among fibrous feeds was confirmed by the present findings. However, the results did not give indications for a more favorable situation in case low-CP-high-fiber tropical diets are used. It was further demonstrated that fibrous feeds differ in their responses to urea addition in apparent OM and fiber degradability. Therefore, using general minimum thresholds for either dietary RDP or ruminal ammonia concentration may be too simplistic. Further screening of fibrous feeds to obtain detailed knowledge of the RDP requirements is needed. This would also help promoting N-efficient ruminant husbandry systems in order to cope with new demands that are increasingly implemented in environmental legislation (e.g., Schwab et al., 2005).

### Conflict of interest statement

The authors declare that the research was conducted in the absence of any commercial or financial relationships that could be construed as a potential conflict of interest.

## Abbreviations

ADF: acid detergent fiber
ADL: acid detergent lignin
CP: crude protein
NDF: neutral detergent fiber
OM: organic matter
Rusitec: Rumen simulation technique
RDP: rumen-degradable protein
SCFA: short-chain fatty acids.
